# New Tetra-Schiff Bases as Efficient Photostabilizers for Poly(vinyl chloride)

**DOI:** 10.3390/molecules22091506

**Published:** 2017-09-09

**Authors:** Dina S. Ahmed, Gamal A. El-Hiti, Ayad S. Hameed, Emad Yousif, Ahmed Ahmed

**Affiliations:** 1 Department of Chemistry, College of Science, Tikrit University, Tikrit 34001, Iraq; dinasaadi86@gmail.com (D.S.A.); ch@sc.nahrainuniv.edu.iq (A.S.H.); 2 Cornea Research Chair, Department of Optometry, College of Applied Medical Sciences, King Saud University, P.O. Box 10219, Riyadh 11433, Saudi Arabia; 3 Department of Chemistry, College of Science, Al-Nahrain University, Baghdad 64021, Iraq; 4 Polymer Research Unit, College of Science, Al-Mustansiriyah University, Baghdad 10052, Iraq; drahmed625@gmail.com

**Keywords:** poly(vinyl chloride), Schiff bases, functional group’s indices, atomic force microscopy, photodegradation, irradiation, viscosity average molecular weight

## Abstract

Three new tetra-Schiff bases were synthesized and characterized to be used as photostabilizers for poly(vinyl chloride) (PVC) films. The photostability of PVC films (40 μm thickness) in the presence of Schiff bases (0.5 wt %) upon irradiation (300 h) with a UV light (λ_max_ = 365 nm and light intensity = 6.43 × 10^−9^ ein∙dm^−3^∙s^−1^) was examined using various spectroscopic measurements and surface morphology analysis. The changes in various functional groups’ indices, weight and viscosity average molecular weight of PVC films were monitored against irradiation time. The additives used showed photostability for PVC films, with Schiff base **1** being the most effective additive upon irradiation, followed by **2** and **3**. The atomic force microscopy (AFM) images for the PVC surface containing Schiff base **1** after irradiation were found to be smooth, with a roughness factor (*R*q) of 36.8, compared to 132.2 for the PVC (blank). Several possible mechanisms that explain PVC photostabilization upon irradiation in the presence of tetra-Schiff bases were proposed.

## 1. Introduction

Polymeric materials are produced in huge quantity and are used in everyday applications. Polyethylene, polypropylene and poly(vinyl chloride) (PVC) are the most produced and used polymers as plastics. The molecular structure of polymeric materials is unique in controlling their chemical, physical and mechanical properties [1,2]. Poly(vinyl chloride) can exit in two common forms, known as rigid and flexible, and has various industrial applications [3]. The rigid PVC can be used in water pipes, windows, doors, synthetic floor tiles, plumbing fittings, phonograph records, and credit cards [4]. The soft PVC can be used as a replacement for rubber in shower curtains, raincoats, and packaging films [4]. Photooxidation can lead to the irreversible deterioration of PVC polymeric materials [5]. Irradiation of PVC with a UV light (250–350 nm) for a long period can lead to a change in color, loss of mechanical and physical properties, reduction in transparency and the formation of cracks and deposits on the surface [6,7,8]. In addition, the PVC photodegradation process can lead to dehydrochlorination and the formation of conjugated double bonds [9]. This is possibly due to impurities during the synthesis process [10], average molecular weight reduction and crosslinking [11,12]. Therefore, the photostability of PVC has to be enhanced for outdoor applications, where the polymeric materials are exposed to light and high temperatures. Various additives at low concentration have been used to enhance the PVC photostability upon UV irradiation [4]. The most common additives include inorganic salts [13,14,15,16], heterocycles [17,18,19,20,21,22,23] and aromatics [24,25,26,27,28]. Schiff bases [29,30,31] and titanium dioxide were found to be the most efficient additives for the photostabilization of PVC [32,33,34].

In this study, we report the successful synthesis of three tetra-Schiff bases, using a simple and efficient procedure, and investigate their PVC photostabilization efficiency as a continuation of our own interest in polymeric materials [35,36,37,38,39,40,41]. The synthesized Schiff bases have been proven to be efficient stabilizers against the photodegradation of PVC samples.

## 2. Results and Discussion

### 2.1. Synthesis and Characterization of Schiff Bases ***1**–**3***

**Scheme 1:**
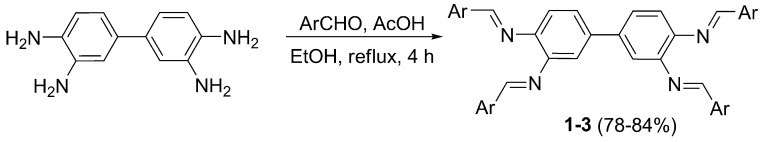
Synthesis of Schiff bases **1**–**3**.

Schiff bases **1**–**3** were synthesized based on literature procedures that have been used for the production of **1** [42,43]. Condensation of biphenyl-3,3′,4,4′-tetraamine and excess aromatic aldehydes (four mole equivalents), namely 2-hydroxybenzaldehyde, 3-hydroxybenzaldehyde and 4-nitrobenzaldehyde, in ethanol in the presence of acetic acid as a catalyst under reflux for 4 h, gave the corresponding Schiff bases **1**–**3** in 78–84% yields (Scheme 1). The physical properties of **1**–**3** and their elemental analyses are presented in Table 1.

**Figure 1:**
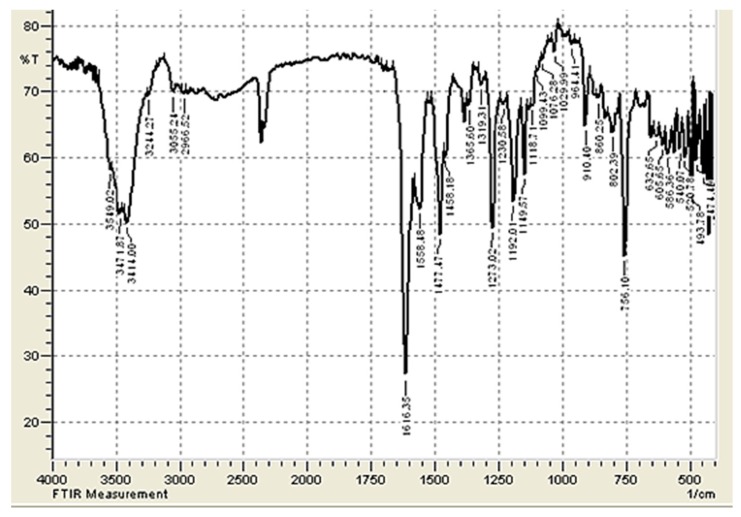
FT-IR spectrum of Schiff base **1**.

The FT-IR spectra of **1**–**3** show the presence of intense absorption bands at 1600−1624 cm^–1^ and can be attributed to the CH=N bonds (Table 2). They also indicate the absence of NH_2_ group absorption. As an example, Figure 1 shows the FT-IR spectrum for Schiff base **1**.

The ^1^H-NMR spectra for **1**–**3** show singlet signals that resonate within the 9.05–8.01 ppm region due to the CH=N protons. They also show all the aromatic protons with the expected multiplicity and chemical shifts (Table 3).

### 2.2. Photodegradation of PVC Films by FT-IR Spectroscopy

**Figure 2:**
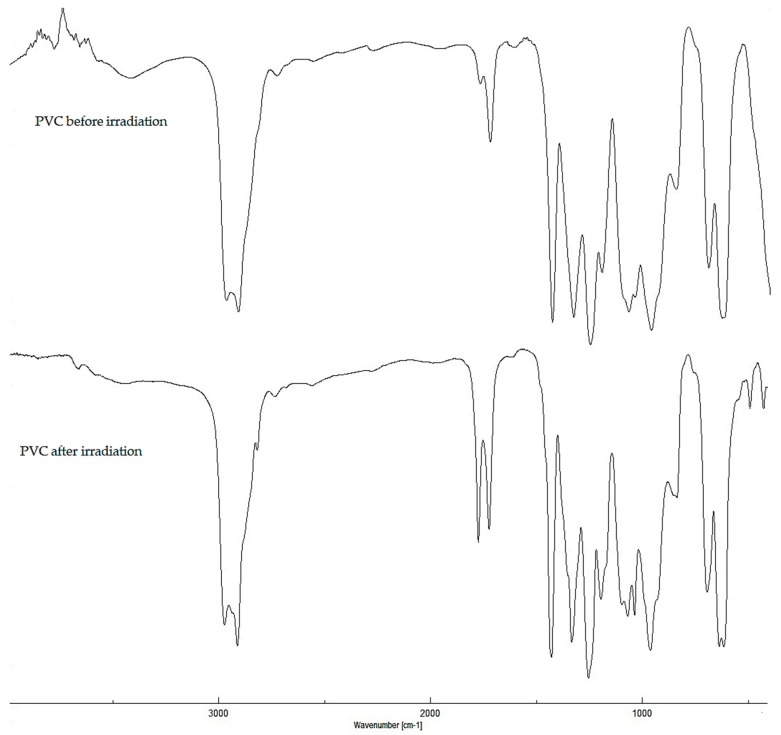
FT-IR spectrum of poly(vinyl chloride) (PVC) films before and after irradiation.

Previous reports show that the most effective concentration of Schiff bases to be added to stabilize PVC was 0.5 wt % [21]. Therefore, Schiff bases **1**–**3** (0.5 wt %) were used as additives to improve the photostability of PVC polymeric materials. The PVC films (40 μm thickness) were irradiated with UV light (λ_max_ = 365 nm and light intensity = 6.43 × 10^−9^ ein∙dm^−3^∙s^−1^) for up to 300 h. The FT-IR spectroscopy (400–4000 cm^−1^) was used to evaluate the changes in certain functional groups within the PVC films. The photostability of PVC films was studied by monitoring the changes in intensity for polyene (1602 cm^−1^), carbonyl (1722 cm^−1^), and hydroxyl (3500 cm^−1^) peaks, as compared to a standard peak (1328 cm^−1^) upon irradiation [44]. The FT-IR spectra of pure PVC films before and after irradiation (300 h) are shown in Figure 2.

**Figure 3:**
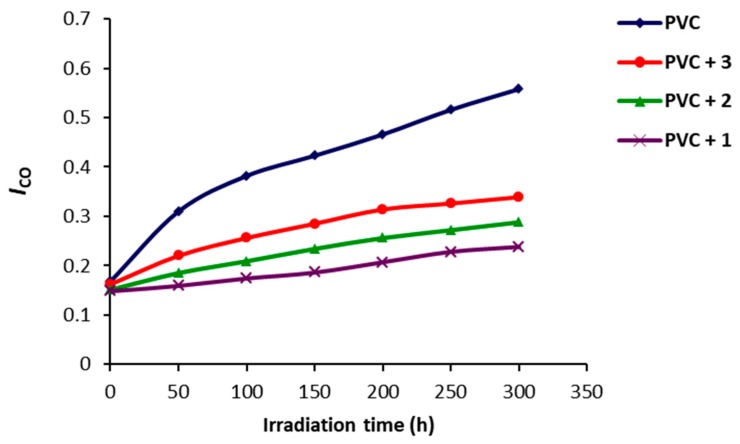
Effect of irradiation of PVC films on *I*_CO_.

**Figure 4:**
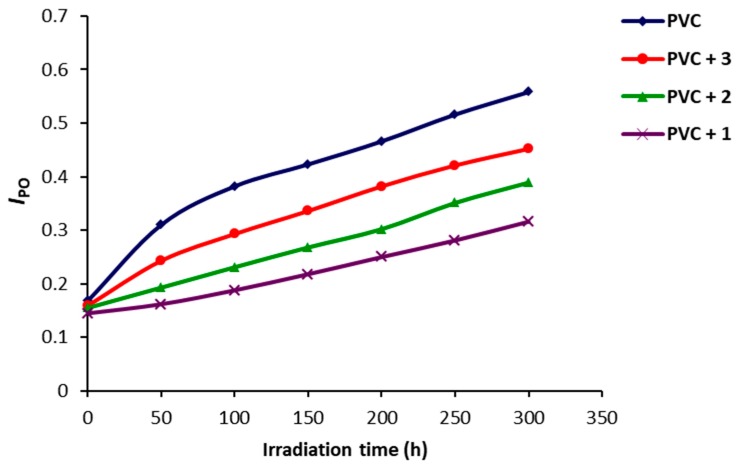
Effect of irradiation of PVC films on *I*_PO_.

**Figure 5:**
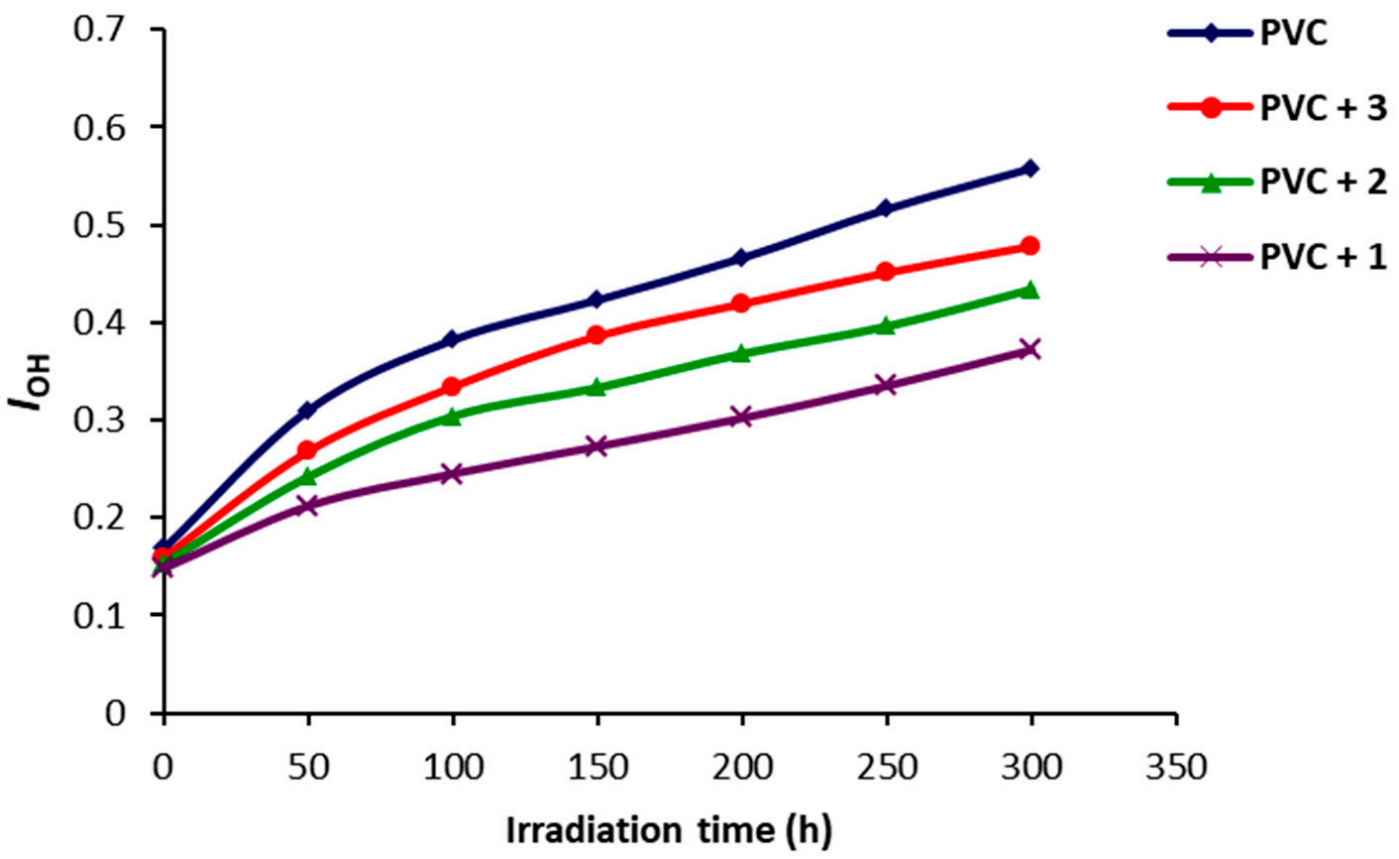
Effect of irradiation of PVC films on *I*_OH_.

The carbonyl (*I*_CO_), polyene (*I*_PO_) and hydroxyl (*I*_OH_) indices were calculated and plotted against irradiation time (Figure 3, Figure 4 and Figure 5). Such indices were lower for the PVC films containing Schiff bases **1**–**3** compared to the ones for the blank PVC. Clearly, Schiff bases **1**–**3** and in particular **1** could be used as efficient photostabilizers for PVC films.

### 2.3. Photodegradation of PVC Films by Weight Loss

**Figure 6:**
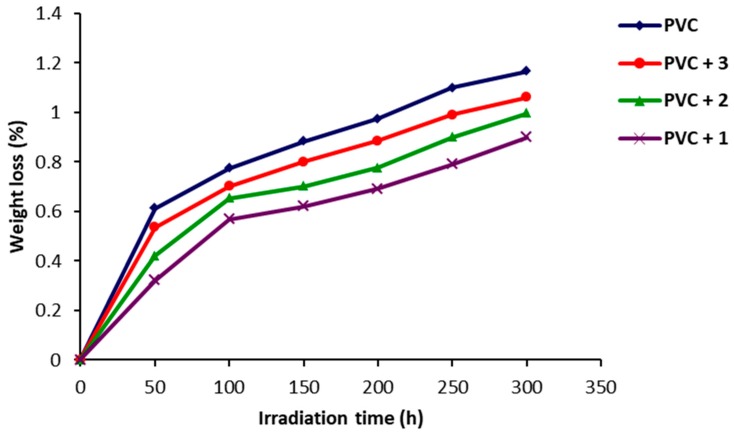
Effect of irradiation of PVC films on weight loss (%).

Dehydrochlorination, elimination of hydrochloride (HCl), is a major cause of PVC photodegradation and leads to weight loss [45]. The degree of photodegradation of PVC can be measured from the percentage of PVC weight loss after irradiation. The effect of irradiation time on the percentage of PVC films weight loss is shown in Figure 6. Clearly, the weight loss in PVC was lower in the presence of Schiff bases **1**–**3** compared to the blank PVC film. Schiff base **1** was the most efficient additive since it could act as an effective radical scavenger, possibly due to the presence of the *ortho*-hydroxyl group, which increases the stability through resonance. During irradiation of PVC films containing Schiff bases, not only was weight loss reduced but the discoloration was less noticeable [46].

### 2.4. Photodegradation of PVC Films by Variation in Molecular Weight

**Figure 7:**
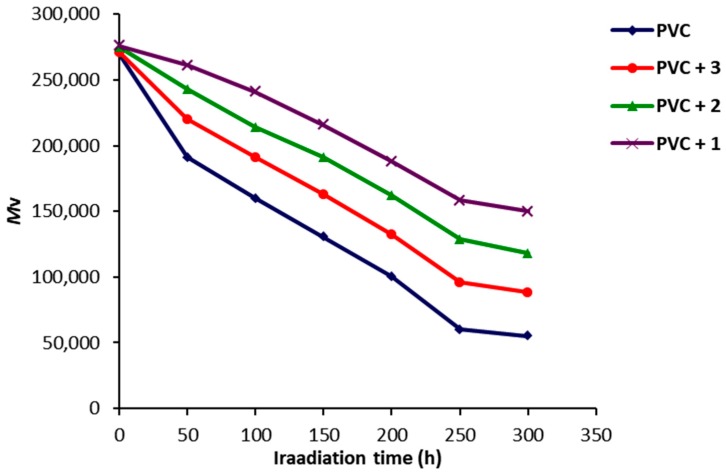
Effect of irradiation of PVC films on ${\bar{M}}_{V}$.

The Mark–Houwink equation [44,47,48] was used to calculate the viscosity average molecular weight $\left({\bar{M}}_{V};\;g/\mathrm{mol}\right)$. Viscosity average molecular weight is directly proportional to the intrinsic viscosity [*η*] [49]. The variation in ${\bar{M}}_{V}$ for PVC films containing **1**–**3** (0.5 wt %) during the irradiation process was measured (Figure 7). The decrease in ${\bar{M}}_{V}$ was higher for the blank PVC film compared to the ones containing **1**–**3**, in which Schiff base **1** was again the most efficient additive. The reduction in ${\bar{M}}_{V}$ during the irradiation process may be due to the degradation of PVC chains producing low molecular weight polymeric chains [50,51].

**Figure 8:**
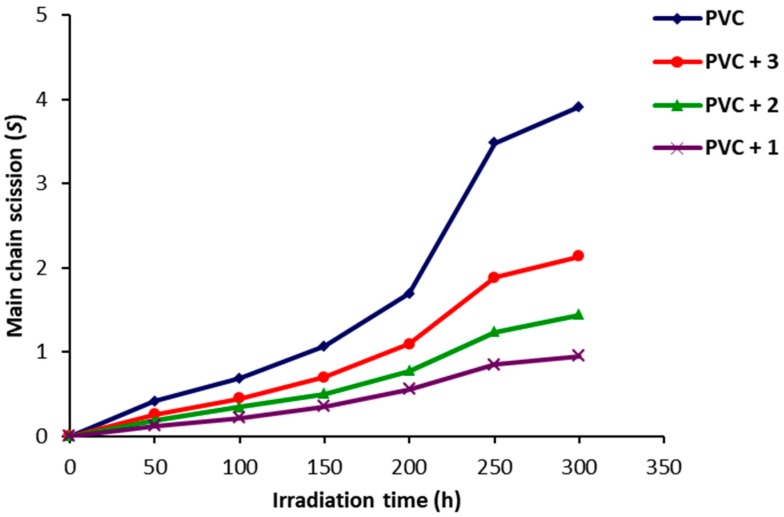
Effect of irradiation of PVC films on *S*.

During the photolysis process, insoluble residue in tetrahydrofuran (THF) was observed and was believed to be due to the PVC chains branching or crosslinking [52]. To confirm this conclusion, the average chain scission (*S*) was calculated using Equation (1) and plotted against irradiation time (Figure 8). The *S* value is dependent on the viscosity average molecular weight at irradiation time 0 (${\bar{M}}_{V,O})$ and *t* (${\bar{M}}_{V,t}$). The results indicate that the degree of branching and/or crosslinking was higher for the PVC (blank) compared to the ones containing Schiff bases. The *S* values for the PVC film containing Schiff base **1** were minimal.

(1) $$S={\bar{M}}_{V,O}/{\bar{M}}_{V,t}-1$$

**Figure 9:**
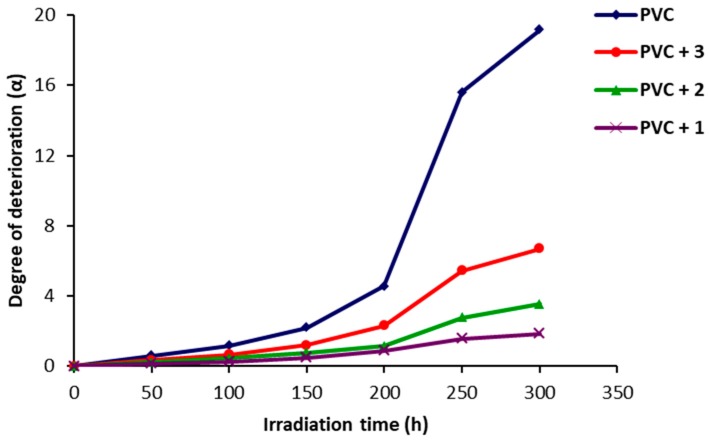
Effect of irradiation of PVC films on α.

At the initial stage of PVC photodegradation, weak bonds, randomly distributed along the polymeric chains, break dawn rapidly. The degree of PVC photodegradation is directly proportional to the degree of deterioration (α). The α values were calculated using Equation (2) from ${\bar{M}}_{V}$, *S* and molecular weight (*m*). The increase in α for the PVC samples was very low in the first 150 h and became sharp afterwards (Figure 9). The α values for the PVC samples containing **1**–**3** were very low compared to the ones obtained for the blank PVC sample.

(2) $$\alpha =m\times S/{\bar{M}}_{\text{V}}$$

### 2.5. Surface Morphology of PVC Films

**Figure 10:**
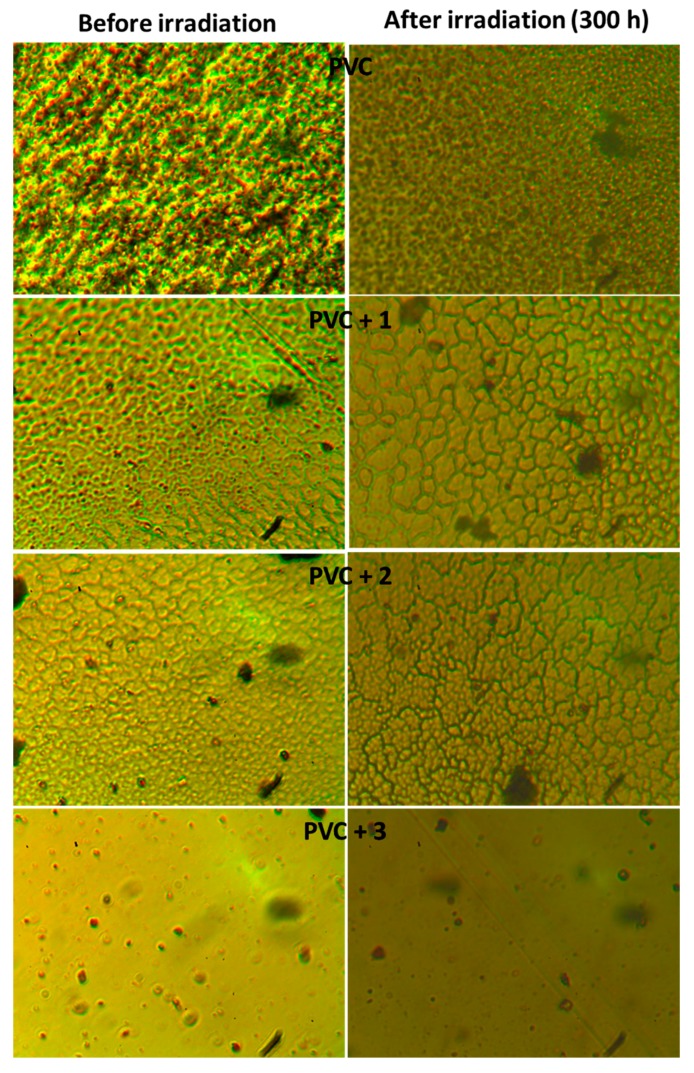
Effect of irradiation of PVC films on surface morphology.

The surface morphology provides information about defects, roughness, and irregularity within the polymeric materials [53]. Also, it can detect the changes in polymer surface upon irradiation where decomposition takes place as chain scission [54]. The surface morphology images for PVC films before and after irradiation (300 h) are shown in Figure 10. Compared to before irradiation, the PVC (blank) image shows the presence of grooves, white spots and cracks on the surface following irradiation. Also, the surface was rough and underwent a color change. Such surface irregularity is a result of photodegradation of PVC due to dehydrochlorination. Comparatively, the images for PVC films containing Schiff bases, in particular **1,** were nearly smooth and have only a few white spots. Clearly, **1**–**3** can inhibit elimination of HCl and enhance the photostability of PVC films upon irradiation.

**Figure 11:**
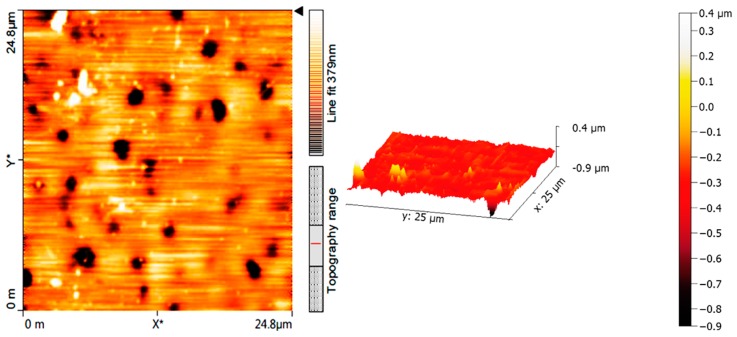
2D and 3D atomic force microscopy (AFM) images of PVC film (blank) after irradiation (300 h).

**Figure 12:**
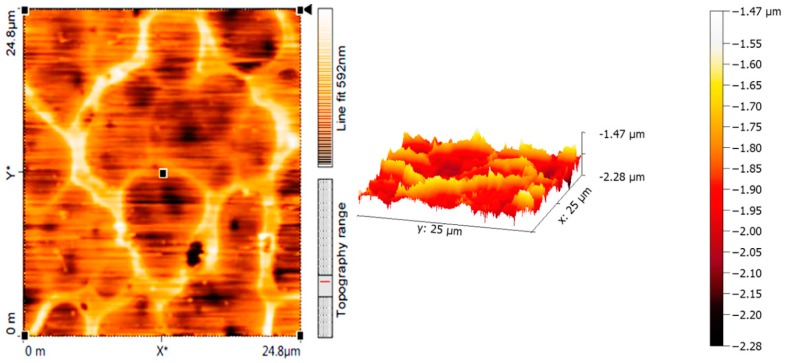
2D and 3D AFM images of PVC film containing **1** after irradiation (300 h).

Atomic force microscopy (AFM) provides two- and three-dimensional images for sample surface and does not require a vacuum environment. It is a simple technique that can be applied in various fields [55]. The 2D and 3D AFM images for the PVC film (bank) which contain Schiff base **1** (0.5 wt %) after irradiation (300 h) are shown in Figure 11 and Figure 12, respectively. The AFM images for the surface of the PVC film (area = 5.0 × 5.0 μm^2^) containing Schiff base **1** after irradiation show it to be very smooth with a low roughness factor (*R*q = 36.8 nm). Comparatively, the surface of the PVC (blank) film was very rough (*R*q = 132.2). The rough surface of the blank PVC film after irradiation could be due to bonds breaking [56] and/or dehydrochlorination [57]. Dehydrochlorination of PVC usually takes place at high temperatures [57].

### 2.6. Proposed Mechanisms of PVC Film Photostabilization

**Scheme 2:**
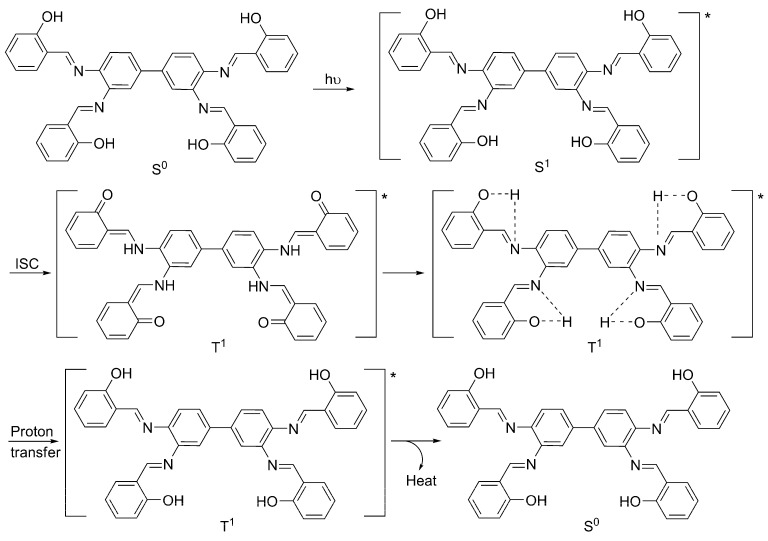
PVC photostabilization via a proton transfer and intersystem conversion in the presence of **1**. * represents the excited state.

The synthesized Schiff bases **1**–**3** act as photostabilizers for PVC films upon irradiation with a UV light. The results show that the efficiency of such additives follows the order **1** > **2** > **3**. Various mechanisms could be suggested to explain the role that Schiff bases **1**–**3** play in the photostabilization of PVC films. The performance of compounds **1**–**3** varies in their stabilization effect on PVC films due to the nature and position of substituents on the aryl rings. Schiff base **1** was the most efficient additive to stabilize PVC films. This is possibly due to the hydroxyl groups at the *ortho*-positions of the aryl rings. Schiff bases can dissipate the UV radiation into harmless heat via a proton transfer for the Schiff base singlet excited state (S_1_ *) and internal conversion (IC), followed by proton transfer. Also, it is possible that the UV energy could be released as harmless heat via intersystem conversion (ISC) of the additive S_1_ * state to the triplet excited state (T_1_ *), followed by a proton transfer, followed by conversion to the ground state (S_0_) [19,30]. Scheme 2 represents the possibility of PVC photostabilization via a proton transfer and ISC in the presence of Schiff base. For most photochemical reactions, only the lowest S_1_ * and T_1_ * states have to be considered for the initiation step [58]. Excited singlet states are short-lived species with a lifetime in the range 10^−9^–10^−6^ s. Photodegradation of polymers often proceeds from T_1_ * states which have a longer lifetime than S_1_ * states, since the return to a S_0_ state is spin-forbidden [58]. The bond breaking within polymeric chains only takes place when the bond dissociation energy is lower than the excitation energy, or when the excited state is a repulsive one.

**Scheme 3:**
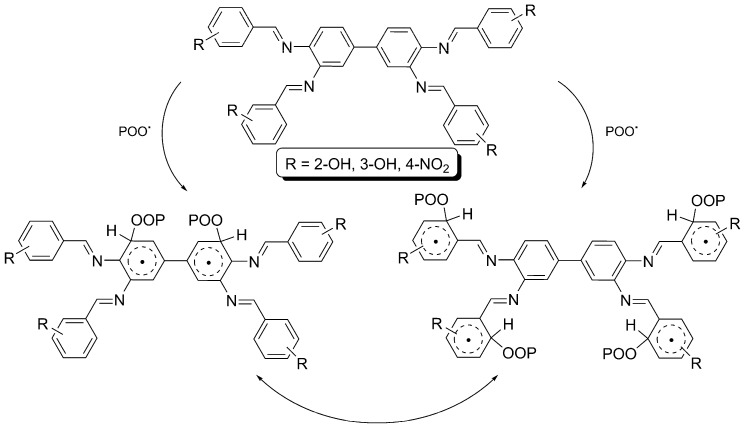
PVC photostabilization via a radical scavenger in the presence of **1**–**3**.

Schiff bases **1**–**3** could act as radical scavengers in the presence of a chromophore (POO^•^) to stabilize the PVC. When excited, a stable complex between the chromophore and Schiff base could be formed, in which energy can be transferred. The stable charge transfer complex would be stabilized via resonance of aryl rings within the Schiff bases (Scheme 3) [59].

**Scheme 4:**
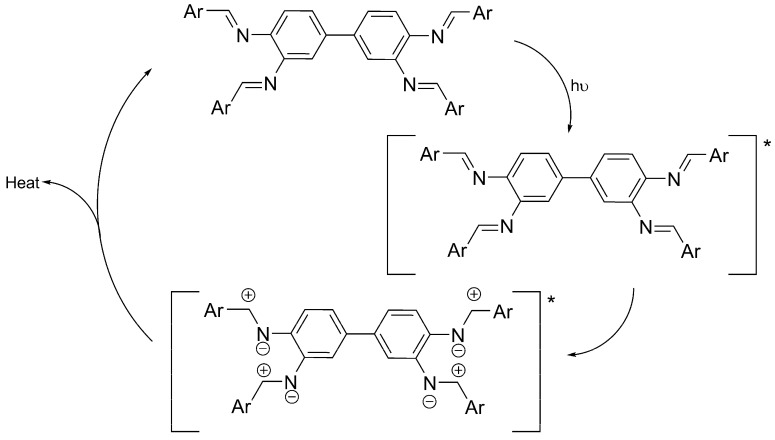
PVC photostabilization of PVC via direct absorption of UV radiation. * represents the excited state.

Tetra-Schiff bases **1**–**3** are electron rich (six aryl rings) and can absorb the UV radiation energy directly and dissipate it into harmless heat energy (Scheme 4). Aromatic compounds commonly act as UV absorbers [60].

**Scheme 5:**
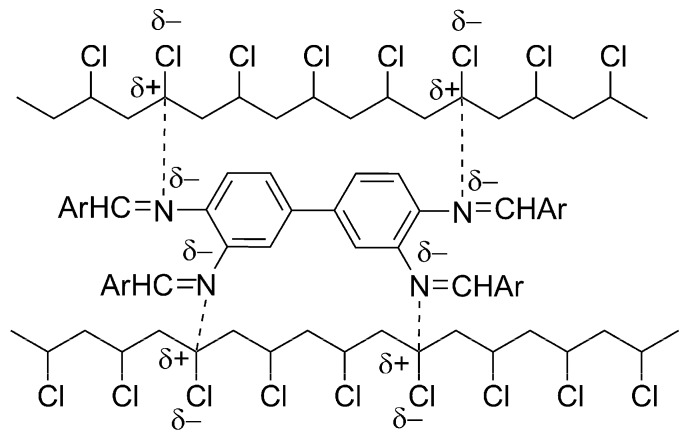
PVC photostabilization via interaction between PVC films and Schiff bases.

The possible attraction between polarized CH=N bonds within Schiff bases **1**–**3** and polarized C-Cl bonds within PVC chains could stabilize PVC (Scheme 5). Such attraction could allow efficient transfer of the PVC excited energy to Schiff bases and then to a harmless level of energy.

## 3. Experimental Section

### 3.1. General

Chemicals were obtained from Sigma-Aldrich Chemical Company (Gillingham, UK). Melting points were recorded on a hot stage Gallenkamp melting point apparatus. The Fourier transform Infrared (FT-IR) spectra (400–4000 cm^−1^) were recorded on a Shimadzu 8400 Spectrophotometer (Shimadzu Cooperation, Kyoto, Japan) using the KBr disk technique. Proton nuclear magnetic resonance (^1^H-NMR) spectra (400 MHz) were recorded on a Bruker DRX400 NMR Spectrometer (Bruker, Zürich, Switzerland) in DMSO-*d*_6_ related to tetramethylsilane. The elemental analyses were performed on the Vario EL III Elementar instrument. An accelerated weather-meter QUV tester (Q-Panel Company, Homestead, FL, USA) was used to irradiate PVC films at room temperature with a UV light (λ_max_ = 365 nm and light intensity = 6.43 × 10^−9^ ein∙dm^−3^∙s^−1^). The morphology images of PVC films were recorded on Meiji Techno Microscope (Meiji Techno, Tokyo, Japan). Atomic force microscopy (AFM) images were recorded on the Veeco instrument (Veeco Instruments Inc., Plainview, New York, NY, USA).

### 3.2. Synthesis of Schiff Bases ***1**–**3***

A mixture of biphenyl-3,3′,4,4′-tetraamine (2.14 g, 10.0 mmol) and appropriate aromatic aldehyde (40.0 mmol) in anhydrous ethanol (25 mL) in the presence of glacial acetic acid (0.5 mL) was stirred magnetically under reflux for 4 h. The mixture was left to cool down to room temperature and the solid obtained was filtered, washed with ethanol and dried to give pure **1**–**3** in 78–84% yields.

### 3.3. PVC Films Preparation

The PVC films were prepared using a solution-cast technique at room temperature. Schiff bases **1**–**3** were added to PVC at a concentration of 0.5% by weight in THF. The solvent was removed by evaporation at room temperature for 24 h. The thickness of PVC films (40 µm) was fixed using a Digital Vernier Caliper 2610A micrometer (Vogel GmbH, Kevelaer, Germany).

### 3.4. Light Exposure

A UV Light (λ_max_ = 365 nm and light intensity = 6.2 × 10^−9^ ein∙dm^−3^∙s^−1^ nm) was used to irradiate the PVC using an accelerated weather-meter QUV tester (Philips, Saarbrücken, Germany) at room temperature. The PVC films were rotated occasionally to ensure that the incident light intensity was the same on all sides.

### 3.5. Photodegradation of PVC Films by FT-IR Spectrophotometry

The changes in IR absorption bands for carbonyl (1722 cm^−1^), polyene (1602 cm^−1^) and hydroxyl (3500 cm^−1^) groups were monitored and compared to a reference peak (1328 cm^−1^). The indices for carbonyl (*I*_C=O_), polyene (*I*_C=C_) and hydroxyl (*I*_OH_) groups were calculated using Equation (3). The functional group index (*I_s_*) is dependent on both the absorbance peak during the study (*A_s_*) and that of the reference peak (*A_r_*) [44].

(3) $$I_{s}=A_{s}/A_{r}$$

### 3.6. Photodegradation of PVC Films by Weight Loss

The weight loss percentage of PVC samples during the irradiation process was calculated by the weight of the PVC sample before (*W*_1_) and after irradiation (*W*_2_) using Equation (4).

(4) $$Weight\;loss\;\%=\left[\left(W_{1}-W_{2}\right)/W_{1}\right]\times 100$$

### 3.7. Photodegradation of PVC Films by Viscometry Method

The Mark–Houwink relation, Equation (5), was used to calculate the PVC films’ average molecular weight (${\bar{M}}_{V}^{\alpha }$) in solution (g/100 mL) using an Ostwald U-tube viscometer [50]. ${\bar{M}}_{V}^{\alpha }$ is highly dependent on the intrinsic viscosity [*η*] of PVC and constants (*α* and *K*) related to the polymer–solvent system.

(5) $$\left[\eta \right]=K{\bar{M}}_{\text{V}}^{\alpha }$$

The relative viscosity (η_re_) at a flow time *t*_0_ (pure solvent) and specific viscosity (η_sp_) were calculated using Equations (6) and (7) in which the flow time for pure solvent (THF) and PVC sample was *t*_0_ and *t*, respectively.

(6) $$\eta_{sp}=\eta_{rel}-1$$

(7) $$\eta_{rel}=t/t_{0}$$

The intrinsic viscosity was calculated using Equation (8) in which *C* is the PVC concentration in THF (g/100 mL).

(8) $$\left[\eta \right]=(\sqrt{2}/c)\;{\left(\eta_{sp}-\ln\eta_{rel}\right)}^{1/2}$$

Molecular weight of PVC samples was calculated from [*η*] using Equation (9).

(9) $$\left[\eta \right]=1.38\times 10^{-4}\;{\bar{M}}_{\text{V}}{}^{0.77}$$

## 4. Conclusions

New tetra-Schiff bases which are highly aromatic were synthesized and characterized using various techniques. The photostability of PVC films was increased in the presence of new Schiff bases. The Schiff bases act as photostabilizers for PVC through direct absorption of the UV radiation, proton transfer and intersystem crossing, radical scavengers or an interaction with PVC chains. The photostabilization activity of PVC films containing additives follows the order **1** > **2** > **3**.

## Figures and Tables

**Table 1:** Physical properties and elemental analyses for Schiff bases **1**–**3**.

Schiff Base	Ar	Color	Yield (%)	Mp (°C)	Calcd. (Found; %)		
					C	H	N
**1**	2-HOC_6_H_4_	Deep orange	84	234–236	8.88 (8.92)	4.79 (4.85)	76.17 (76.22)
**2**	3-HOC_6_H_4_	Light brown	80	207–209	8.88 (8.91)	4.79 (4.83)	76.17 (76.19)
**3**	4-NO_2_C_6_H_4_	Dark red	78	274–276	15.01 (15.23)	3.51 (3.56)	64.34 (64.40)

**Table 2:** Some FT-IR spectral data for Schiff bases **1**–**3**.

Schiff Base	FR-IR (υ, cm^−1^)			
	OH	CH=N	C=C (Ar)	C-N
**1**	3414	1616	1558	1273
**2**	3417	1600	1527	1273
**3**	-	1624	1516	1342

**Table 3:** ^1^H-NMR spectral data for Schiff bases **1**–**3**.

Schiff Base	^1^H-NMR (400 MHz: DMSO-*d*_6_, δ, ppm, *J* in Hz)
**1**	9.17 (s, exch., 4 H, OH), 9.08 (s, 4 H, CH), 7.93 (s, 2 H, Ar), 7.71 (d, *J* = 8.2 Hz, 4 H, Ar), 7.64 (d, *J* = 8.5 Hz, 2 H, Ar), 7.44 (t, *J* = 8.2 Hz, 4 H, Ar), 7.25 (d, *J* = 8.5 Hz, 2 H, Ar), 7.00 (t, *J* = 8.2 Hz, 4 H, Ar), 6.99 (d, *J* = 8.2 Hz, 4 H, Ar)
**2**	8.08 (s, exch., 4 H, OH), 8.01 (s, 4 H, CH), 7.63 (s, 2 H, Ar), 7.53 (d, *J* = 8.1 Hz, 4 H, Ar), 7.48 (d, *J* = 8.5 Hz, 2 H, Ar), 7.48 (s, 4 H, Ar), 7.35 (d, *J* = 8.5 Hz, 2 H, Ar), 7.18 (t, *J* = 8.1 Hz, 4 H, Ar), 6.95 (d, *J* = 8.1 Hz, 4 H, Ar)
**3**	8.61 (s, 4 H, CH), 8.32 (d, *J* = 8.3 Hz, 8 H, Ar), 8.05 (d, *J* = 8.3 Hz, 8 H, Ar), 7.70 (s, 2 H, Ar), 7.50 (d, *J* = 8.4 Hz, 2 H, Ar), 7.35 (d, *J* = 8.4 Hz, 2 H, Ar)
